# Association of systemic immune inflammation index with depression among adult type 2 diabetic patients in a tertiary hospital, Ethiopia, 2022

**DOI:** 10.3389/fendo.2025.1454793

**Published:** 2025-02-21

**Authors:** Seid Yimam Ali, Mohammed Ibrahim Sadik, Ahmed Muhye Seid, Awol Hassen Mohammed, Ahmed Adem Yimam, Mekonin Meskelu Shegere, Tesfaneh Shimels Ayele, Abdulmalik Jeben Wako, Mohamedaman Mohamedsied Ibrahim

**Affiliations:** ^1^ School of Medicine, Institute of Health, Jigjiga University, Jigjiga, Somali, Ethiopia; ^2^ Department of Biomedical Sciences, Institute of Health, Jimma University, Jimma, Oromia, Ethiopia; ^3^ Department of Public Health, College of Medicine and Health Sciences, Dire Dawa University, Dire Dawa, Ethiopia; ^4^ School of Medicine, College of Medicine and Health Science, Wollo University, Dessie, Amhara, Ethiopia; ^5^ Department of Internal Medicine, College of Medicine and Health Science, Jigjiga University, Jigjiga, Somali, Ethiopia; ^6^ Department of Public Health, College of Medicine and Health Science, Debre Tabor University, Debre Tabor, Amhara, Ethiopia; ^7^ Department of Biomedical Science, College of Medicine and Health Science, Arsi University, Asella, Oromia, Ethiopia; ^8^ Department of Surgery, Faculty of Medicine and Health Science, University of Bosaso, Garowe, Puntland, Somalia

**Keywords:** T2DM, depression, SII, khat, socioeconomic factors

## Abstract

**Background:**

The inflammatory and hormonal aspects of T2DM can influence the development or worsening of depressive symptoms. While most of the burden had due consideration, the mental health impact of T2DM such as depression is often unnoticed, undiagnosed, or untreated. Despite some studies exploring depression in Ethiopian T2DM patients, none have investigated the predictive role of the Systemic Immune inflammation Index (SII) in depression.

**Objective:**

This study aimed to determine the prevalence of depression and its association with the Systemic Immune Inflammation Index (SII), socio-economic factors, and behavioral predictors among adult T2DM patients at Jigjiga University Comprehensive Specialized (JJU CS) Hospital.

**Methods and materials:**

A hospital-based cross-sectional study was conducted from Oct 3 to Nov 13/2022 at JJUSH. Depression was assessed by using the Patient Health Questionnaire (PHQ-9). A complete blood count was done to calculate SII. Data entry was conducted using Epidata version 4.6 and subsequently analyzed in SPSS-V-26. Multiple logistic regression with the backward elimination method was performed. Variables with a p-value < 0.05 were considered statistically significant at a 95% CI

**Results:**

A total of 263 T2DM patients were recruited in the study. Of these, 134 individuals (51%) were male. The overall prevalence of depression was 47.1%. Of the 263 enrolled T2DM patients, the majority, 142 (54%) had elevated SII. A multivariable logistic regression analysis indicated that high SII (AOR= 2.76), current Khat chewers (AOR= 2.83), ex-Khat chewers (AOR= 4.12), and female sex (AOR= 2.68) were identified independent predictors of depression.

**Discussion:**

There was a high prevalence of depression among T2DM patients on follow-up at JJUSH. As well, SII, Khat chewing, and female sex were identified as predictors of depression. Therefore, relevant stakeholders should work towards control of systemic inflammation, avoidance of risky behaviors such as khat chewing, and promotion of healthy behavior particularly among females.

## Introduction

Type 2 Diabetes Mellitus (T2DM) is a widespread and complex metabolic disorder characterized by insulin resistance and elevated blood glucose levels (1, 2). According to the International Diabetes Federation (IDF) in 2021, 537 million (10.5%) people worldwide have diabetes, with T2DM accounting for more than 90% of all cases globally (3). It has emerged as a global health challenge, particularly in low- and middle-income countries undergoing rapid urbanization and lifestyle changes (4). T2DM can lead to various debilitating complications, such as diabetic retinopathy, nephropathy, neuropathy, and an increased risk of cardiovascular diseases (5).

Depression is a significant global public health concern, affecting approximately 280 million (3.8%) people worldwide. It is characterized by persistent sadness, hopelessness, and a range of emotional, cognitive, and physical symptoms (6). It is ranked third in the global burden of disease and projected to become the leading cause by 2030 (7). The development of depression is influenced by a complex interplay of genetic, biological, environmental, and psychological factors, including chronic illness like T2DM (8).

T2DM and depression share overlapping risk factors, such as sedentary lifestyles, poor dietary choices, and obesity (9). These conditions often coexist, influencing each other reciprocally (10). Chronic inflammation, insulin resistance, and hormonal imbalances in T2DM can contribute to the onset or exacerbation of depressive symptoms (11, 12). The psychological burden of managing T2DM, including monitoring blood sugar levels and coping with the fear of complications, also heightens the risk of depression (13). Biological mechanisms underpinning the connection between depression and T2DM include neurotransmitter dysregulation, chronic stress affecting the Hypothalamic-Pituitary-Adrenal (HPA) axis, and reductions in Brain-Derived Neurotrophic Factor (BDNF), all contributing to mood disorders (14–16).

Chronic systemic inflammation (CSI) is a significant factor linking T2DM and depression (17). The Systemic Immune Inflammation Index (SII), a marker of CSI, has been associated with treatment-resistant depression and elevated proinflammatory biomarkers in individuals with depressive symptoms (18). Proinflammatory biomarkers are often elevated in individuals with depressive symptoms (19, 20). Stressors trigger changes in the immune system, affecting both innate and adaptive immunity (21). Neutrophils, platelets, lymphocytes, and proinflammatory cytokines are involved in the complex interaction between inflammation and depression (21–23). This suggests that inflammation may be a key mechanism in the relationship between these two conditions.

Despite the high burden of both T2DM and depression, the mental health aspect is often ignored in clinical settings (24, 25). Depression as a comorbid condition imposes a multidimensional burden, affecting physical, mental, and socioeconomic aspects of health. Elevated SII in T2DM patients is linked to a higher risk of developing depression through chronic systemic inflammation (26, 27). While some studies have explored depression prevalence and related factors in Ethiopian T2DM patients, none have investigated the predictive role of the Systemic Immune-Inflammatory Index (SII) in depression, despite its potential to positively impact both depression and cardiovascular disease risk in T2DM patients (11, 28, 29).

Khat, a plant native to East Africa and the Arabian Peninsula, is known for its stimulant effects due to cathinone (30). Its use is prevalent in Ethiopia and has been linked to mental health issues such as anxiety and depression (31, 32). Although the World Health Organization classifies khat as a substance with potential for psychological dependence (33, 34), its exact impact on depression in T2DM patients remains under-researched. This study aims to explore the relationship between khat use, systemic inflammation, and depression in Ethiopian T2DM patients.

## Methodology

### Study setting and period

The study was conducted at JJU CS Hospital, situated in Jigjiga, the capital city of the Somali Region in Eastern Ethiopia, from October 3 to November 13, 2022. The hospital, established in 2017, provides medical services to the local community, as well as individuals from Somalia, offering specialized medical care for various health conditions, including diabetes. During the data collection period, there were 474 T2DM patients actively participating in regular follow-up appointments.

### Study design

A Hospital-based cross-sectional study design was employed.

### Study population and sampling techniques

The sample size was determined using the single population proportion formula, taking into account a prevalence of depression in T2DM patients from a previous study conducted in Harar, Eastern Ethiopia, which was 48.9% (29). The calculation considered a 95% confidence level, a 5% margin of error, and an estimated total population of 712 during the study period. Ultimately, 278 T2DM patients aged 18 years and above with a history of T2DM lasting for at least one year from the time of diagnosis, and who were receiving diabetic follow-up care at JJUSH were selected for the study. For the selection of study units, a systematic sampling technique was used and every other consenting patient visiting the clinic was enrolled. Consequently, 263 participants willingly participated, resulting in a response rate of 94.6%.

#### Exclusion criteria

Excluded individuals encompassed those with T2DM who were attending their follow-up for the second time during the data collection period, those with a pre-existing history of depression before the onset of DM, individuals diagnosed with any mental disorders, those who had experienced the recent death or loss of a close family member or loved one, patients with hypothyroidism, mothers in the peripartum period, individuals currently taking corticosteroids, and those with acute infections.

### Data collection procedures

Data collection involved an interviewer-administered structured questionnaire with four major sections: socioeconomic information, behavioral predictors, depression assessment, and laboratory measurements. Depression was assessed using the Patient Health Questionnaire-9 (PHQ-9), a widely recognized and validated tool for depression screening (35, 36). The PHQ-9 comprises nine questions that assess the frequency of various depressive symptoms over the past two weeks, with each symptom rated on a scale from 0 (not at all) to 3 (nearly every day). Total scores range from 0 to 27, with higher scores indicating greater depression severity (37, 38). Behavioral predictors were assessed using the WHO drug addiction questionnaire and the National Health Interview and Survey (NHIS), modified to align with the study’s objectives (39, 40). The questionnaires were translated into the local languages (Amharic and Somali) to minimize biased responses. A pretest was conducted with 14 T2DM patients at Karamara General Hospital. The necessary modifications were made based on feedback from the pretest before implementation in the main study.

About 3ml of venous blood was collected into a container with EDTA anticoagulant tube. The blood drawn into EDTA-containing tubes was refrigerated at 4°C. Blood was subjected to full blood count measurements using the Fully Automated XN-550 Automated Hematology Analyzer (11). The SII was calculated based on peripheral platelet, granulocyte, and lymphocyte blood counts using the formula: SII = P * N/L, expressed as a ratio (11). The optimal cut-off value for SII was 410×109 cells/L, classifying patients with SII ≤ 410 (×109 cells/L) as having low SII and those with SII > 410 (×109 cells/L) as having high SII (41, 42).

### Operational definitions

Depression: A T2DM patient with a PHQ-9 score of 5 and above is considered to have depression (36, 38).

High SII: A T2DM patient is classified as having a high SII if their SII or N*P/L ratio is greater than 410 (×10^9^ cells/L) (41, 42).

Active substance User: A T2DM patient is designated as an “Active User” if they have used Khat, consumed alcohol at a rate exceeding 14 drinks per week for males and 7 drinks per week for females, or smoked more than 100 cigarettes in their lifetime, and have engaged in these activities in the last 28 days (43–45).

Ex-substance User: Similar to an “Active Substance User,” but they have not engaged in these activities in the last 28 days (43–45).

Non-substance User: A T2DM patient falls into this category if they have not used Khat, consumed alcohol at a rate exceeding 14 drinks per week for males and 7 drinks per week for females, or smoked more than 100 cigarettes in their lifetime, or have not done so in the last 28 days (43–45).

Adherence to physical activity: A T2DM patient is considered adherent to physical activity if they engage in moderate aerobic activity for at least 30–60 min. per day for 3 or more days per week.

### Data processing and analysis

The collected data underwent a rigorous quality control process, including checks for completeness, editing, coding, and entry into EpiData (version 4.6.win.64). Subsequently, the data was exported to SPSS version 26 for statistical analysis. Descriptive statistics, such as mean, frequency, and percentage, were used to summarize the data, and the results were presented using figures and tables. Initially, bivariate analysis identified explanatory variables associated with the outcome variable, using a significance level of P-value less than 0.25. These variables were then included in the multiple logistic regressions with backward elimination method. Independent predictors were determined using multivariable logistic regression analysis, and statistical significance was declared for variables with a P-value less than 0.05 and the strength of associations was determined using adjusted odds ratio (AOR) at 95% CI.

### Ethics approval and consent to participate

Ethical approval for this study was obtained from the Institutional Ethical Review Board of Jimma University, Institute of Health (IRB No. 79/22). Before participating, all individuals provided informed consent, both in written and verbal forms, following a comprehensive explanation of the research’s objectives and procedures. The Chief of Outpatient Services played a crucial role in supervising the consent process for individuals who were unable to read and write. Their presence guaranteed the fair and ethical execution of the procedure, as approved by the Research Ethics Board mentioned earlier. It was emphasized that participation in the study was entirely voluntary, and participants were informed of their right to withdraw or decline participation at any stage without facing any external pressures. To protect confidentiality, numerical codes were utilized instead of names on the questionnaires, preserving the anonymity and privacy of the participants’ responses. Patients found to have severe depression during data collection were referred to the psychiatric department for further assessment and treatment. The study adhered to ethical guidelines and strictly adhered to the ethical principles outlined in the Declaration of Helsinki.

## Results

### Socio economic characteristics

A total of 263 T2DM patients were recruited in the study. Of these, 134 individuals (51%) were male and the average age of the respondents was 50.21 ± 14.81 years. Majority 127 (48.3%) of respondents were found in late adulthood age group (41-60 years) and 182 (69.2%) were married. Three forth 197 (74.9%) of the respondents were urban dwellers and half 132 (50.2%) of respondent’s estimated monthly income were 4-8 thousand ETB (**Table 1**).

**Table 1:** Socio-economic characteristics of adult T2DM patients on chronic follow up at JJU CS Hospital, Ethiopia, 2022 (n=263).

Variables	Category	Frequency (n)	Percent (%)
Age of respondents (years)	18-40	77	29.3
	41-60	127	48.3
	>60	59	22.4
Sex of respondents	Female	129	49.0
	Male	134	51.0
Marital status	Married	182	69.2
	Widowed	27	10.3
	Divorced	23	8.7
	Single	31	11.8
Residence of respondents	Urban	197	74.9
	Rural	66	25.1
Educational status	Illiterate	48	18.5
	Informal	40	15.2
	Primary	75	28.5
	Secondary	63	24
	Higher	37	14.1
Occupation types	Gove-t employee	46	17.5
	Merchant	59	22.4
	Private/NGO	54	20.5
	Daily labor	31	11.8
	Housewife	55	20.9
	Others	18	6.8
Estimated monthly income (Ethiopian Birr)	<4,000	37	14.1
	4-8,000	132	50.2
	9-16,000	72	27.4
	>16,000	22	8.4

### Measurement of behavioral predictors

Among 263 respondents, 105 (39.9%) were Khat chewer. More than half of the respondents 149 (56.6%) had less than optimal physical activity level (**Table 2**).

**Table 2:** Behavioral measurements of adult T2DM patients on chronic follow up at JJU CS Hospital, Ethiopia, 2022 (n=263).

Variables		Category	Frequency (n)	Percent (%)
Khat chewing	Chewer	Active chewer	60	22.8
		Ex-chewer	45	17.1
		Non-chewer	148	60.1
Cigarette smoking	Smoker	Current-smoker	52	19.8
		Ex-smoker	32	12.2
		Non-smoker	179	68.1
Alcohol drinking	Drinker	Current-drinker	45	17.1
		Ex-drinker	22	8.4
		Non-drinker	196	74.5
Exercise	Adherent	Vigorous	21	8.0
		Moderate	93	35.4
	Non-adherent	Mild	20	7.6
		Inactive	129	49.0

### Systemic immune inflammation index

**Figure 1:**
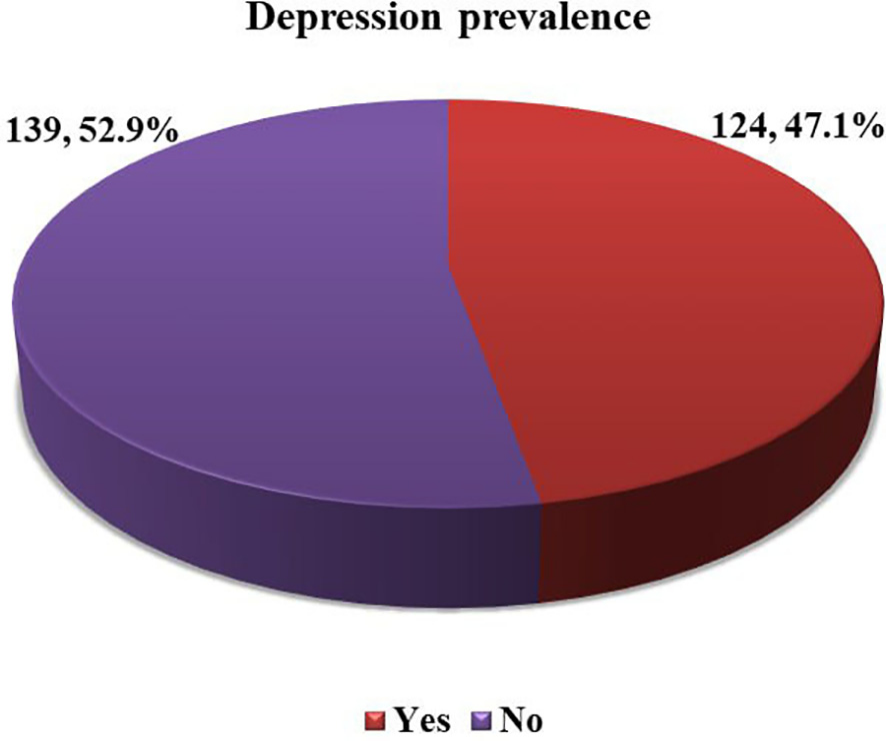
Level of SII among adult T2DM patients on chronic follow up at JJU CS Hospital, Ethiopia, 2022 (n=263).

Of the 263 enrolled T2DM patients, the majority, 142 (54.0%) had elevated SII. The mean value of SII was 584.54 ± 485.4 ×10^9^ cells/L) with a minimum value of 65 and a maximum value 2868 (×10^9^ cells/L) (**Figure 1**).

### Prevalence and severity of depression among diabetic patients

**Figure 2:**
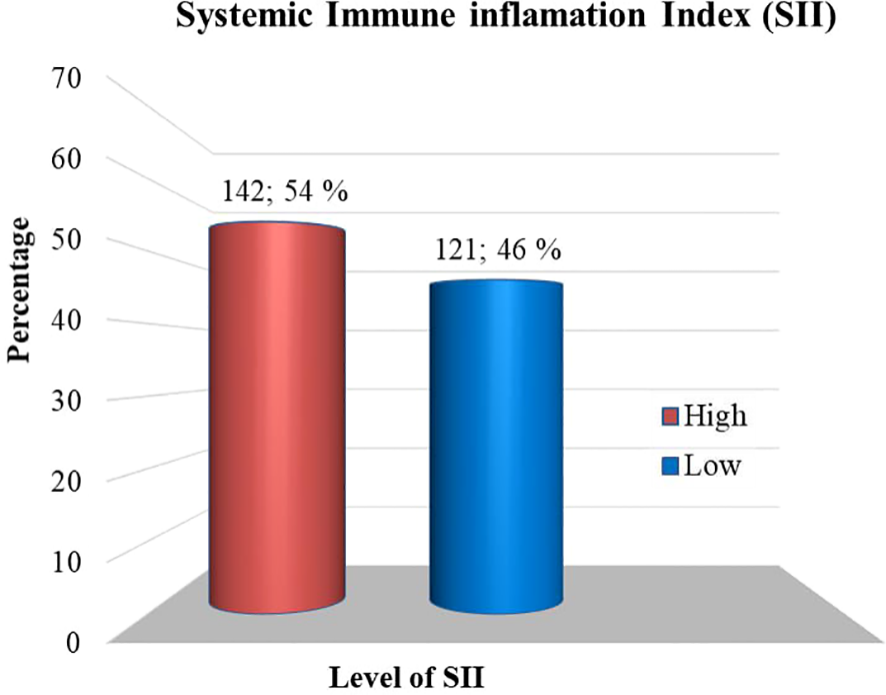
Depression status among adult T2DM patients on chronic follow-up at Jigjiga University Specialized Hospital, Ethiopia, 2022 (n=263).

The study found that the overall prevalence of depression was 124 (47.1%) (95% CI= 41.1, 53.2). Then again, 139 (52.9%) of the participants, had PHQ-9 scores of < 5, indicating that they clinically did not have depression (**Figure 2**).

### Binary and multivariable analysis

In the bivariate analysis, various variables from socio-economic, behavioral, and SII factors were considered. Among these variables, five showed an association with depression among T2DM patients, with a p-value of less than 0.25 in binary logistic regression analysis. These variables, which included age category, female sex, Khat chewing, levels of exercise, and SII, were subsequently included in a multiple logistic regression analysis using the backward elimination method. Among all the variables included in the multiple logistic regression model, three variables were found to be statistically significant at a p-value of less than 0.05. These variables were SII, female sex, current and ex-Khat chewing status, and SII. They were identified as having an independent and significant association with depression among T2DM patients.

Specifically, participants of female sex were 2.7 times more likely to experience depression than their male counterparts (AOR= 2.68; 95% CI= 1.50, 4.80; p= 0.001). It was also identified that current Khat chewers were about three times (AOR= 2.83; 95% CI= 1.41, 5.68; p= 0.003) more likely to experience depression than those non-chewers and ex-Khat chewers also were about four times (AOR= 4.12; 95% CI= 1.88, 9.12; p= 0.000) more likely to experience depression than those non-chewers. Patients with high SII levels were 2.8 times more likely to suffer from depression than those with low SII levels (AOR= 2.76; 95% CI= 1.55, 4.91; p= 0.001).

Some variables that had shown associations in bivariate analysis, such as age group and activity levels, did not maintain a statistically significant association with depression in the final model. Therefore, they were not considered potential independent factors for depression among adult T2DM patients (**Table 3**).

**Table 3:** Bi-variable and multi-variable analysis to determine the independent predictors of depression among adult T2DM on chronic follow up at JJU CS Hospital, Ethiopia, 2022 (n= 263).

Variables	Category	Depression (n, %)		Bivariate analysis		Multivariate analysis	
		No	Yes	COR (CI)	p-value	AOR (CI)	p-value
Age (years)	18-40	52 (67.5)	25 (32.5)	1		1	
	41-60	63 (49.6)	64 (50.4)	2.11 (1.17, 3.81)	.013	1.47 (0.73, 2.96)	.282
	>60	24 (40.7)	35 (59.3)	3.03 (1.50, 6.14)	.002	1.59 (.66, 3.82)	.303
Sex	Female	57 (44.2)	72 (55.8)	1.99 (1.22, 3.26)	.006	**2.68 (1.50, 4.80)**	**.001**
	Male	82 (61.2)	52 (38.8)	1		1	
Educational status	Illiterate	28 (58.3)	20(41.7)	.75 (.32, 1.79)	.521		
	Informal	22 (55.0)	18 (45.0)	.86 (.35, 2.12)	.749		
	Primary	42 (56.0)	33 (44.0)	.83 (.38, 1.83)	.642		
	Secondary	28 (44.4)	35 (55.6)	1.32 (.59, 2.98)	.504		
	Higher	19 (51.4)	18 (48.6)	1			
Occupation of participants	Gov-t employee	27 (58.7)	19 (41.3)	1			
	Merchant	34 (57.6)	25 (42.4)	1.05 (.48, 2.28)	.912		
	Private	21 (38.9)	33 (61.1)	2.23 (1.00, 4.98)	.050		
	Daily labor	18 (58.1)	13 (41.9)	1.03 (.41, 2.59)	.956		
	Housewife	28 (50.9)	27 (49.1)	1.37 (.62, 3.02)	.434		
	Others	11 (61.1)	7 (38.9)	.90 (.30, 2.76)	.860		
Khat chewing	Current-chewer	24 (40.0)	36 (60.0)	2.45 (1.33, 4.50)	.004	**2.83 (1.41, 5.68)**	**.003**
	Ex-chewer	17 (37.8)	28 (62.2)	2.69 (1.36, 5.33)	.005	**4.12 (1.88, 9.12)**	**.000**
	Non-chewer	98 (62.7)	60 (37.3)	1		1	
Cigarate smoking	Current-smoker	25 (48.1)	27 (51.9)	1.22 (.66, 2.27)	.526		
	Ex-smoker	19 (59.4)	13 (40.6)	.77 (.36, 1.67)	.511		
	Non-smoker	95 (53.1)	84 (46.9)	1			
Alcohol drinking	Current-drinker	22 (48.9)	23 (51.1)	1.23 (.64, 2.36)	.529		
	Ex-drinker	11 (50.0)	11 (50.0)	1.19 (.49, 2.84)	.716		
	Non-drinker	106 (54.1)	90 (45.9)	1			
Exercise	Vigorous	14 (66.7)	7 (33.3)	0.42 (.16, 1.11)	.081	0.54 (0.18, 1.63)	.276
	Moderate	59 (63.4)	34 (36.6)	0.49 (0.28, 0.84)	.010	0.70 (0.37, 1.31)	.262
	Mild	7 (35.0)	13 (65.0)	1.57 (0.59, 4.18	.371	1.77 (.56, 5.54)	.330
	Inactive	59 (45.7)	70 (54.3)	1		1	
SBP	<129	72 (56.3)	56 (43.8)	1			
	130-139	37 (49.3)	38 (50.7)	1.32 (.75, 2.34)	.341		
	140-159	20 (48.8)	21(51.2)	1.35 (.67, 2.73)	.404		
	>160	10 (52.6)	9 (47.4)	1.16 (.44, 3.04)	.767		
SII	High	60 (42.3)	82 (57.7)	2.57 (1.56, 4.24)	.000	**2.76 (1.55, 4.91)**	**.001**
	Low	79 (65.3)	42 (34.7)	1		1	

The bold values indicated statistically significant variables.

COR, crude odds ratio; AOD, adjusted odds ratio; CI, confidence interval.

## Discussion

This study aimed to assess the prevalence of depression and examine its association with the Systemic Immune Inflammation Index (SII), socio-economic factors, and behavioral predictors among adult Type 2 Diabetes Mellitus (T2DM) patients in Ethiopia. In this study, elevated SII, Khat chewing, and being female sex were significant and independent predictors of depression among T2DM patients in multivariable logistic regression analysis. To the best of our knowledge, this is the first study in Ethiopia that determines the prediction of elevated SII on depression in T2DM patients. T2DM patients with elevated SII were 2.8 times (AOR = 2.76; 95% CI = 1.55, 4.91) more at risk of depression compared to patients with low SII. This finding was consistent with a large-scale survey conducted in the USA, which reported that, after adjusting for all other variables, high SII remained an independent risk factor for depression among individuals with diabetes (11) (**Figure 1**). The prevalence of chronic systemic inflammation in depression was estimated by another biomarker in a study conducted in the United Kingdom, a quarter of patients with depression show evidence of chronic systemic inflammation (CRP > 3 mg/L) (46). This could be explained by the fact that patients with high SII often have thrombocytosis, neutrophilia, or lymphopenia. SII is a novel inflammatory biomarker based on neutrophil, lymphocyte, and platelet counts (SII = N*P/L) that accurately reflects CSI (47, 48). Neutrophils, which constitute the largest proportion of white blood cells, are important for initiating and modulating immune processes and secrete neutrophil elastase and ROS, which mediate chronic inflammation and may be involved in the development of depression. Lymphocytes are specific inflammatory mediators with regulatory or protective effects and cortisol decreases the number of lymphocytes in the blood. Platelets can be considered a specific first-line inflammatory marker that can bind to leukocytes and the endothelium, influencing the function of inflammatory elements of these cells by secreting inflammatory chemical mediators (49). The increment of pro-inflammatory cytokines and their ability to access CNS interact with neurotransmitter metabolism, neuroendocrine function, and synaptic plasticity, may be involved in the progress of depression (50).

Khat chewing also augments the occurrence of depression among Khat chewers. In this study, ex-Khat chewers were about 4 times (AOR = 4.12; 95% CI = 1.88, 9.12) and current Khat chewers were 3 times (AOR = 2.83; 95% CI = 1.41, 5.68) more likely to have depression compared to non-chewers. This result was in line with the study in Harar (Ethiopia) (29) and Jizan (Saudi Arabia) (51). This might be due to the fact that Khat contains the psychoactive chemical cathinone that has amphetamine-like action in the brain which activates the release of monoaminergic NTs such as dopamine in the limbic system resulting in reward sensations (52). However, after the cessation of Khat, it leads to depression. Similarly, cathinone increases levels of DA results in reduced functioning of D2 receptors in the striatum and dysfunctions in the prefrontal and orbitofrontal cortex – areas that have been shown to play major roles in changes in the reward-value of the outcome. Cathinone also causes white matter abnormalities, lower cortical gray matter volume, and higher striatal volume (52). Khat chewers are more vulnerable to oxidative stress, as shown by increased levels of salivary malonyl-dialdehyde and decreased levels of total antioxidant capacity (53). This results in inflammation and neurodegeneration in the brain- the most vulnerable organ to the damaging effects of ROS due to its high metabolic rate, which plays a role in the pathogenesis of depression (54).

Finally, this study justified that being female carries 2.7 times (AOR = 2.68; 95% CI = 1.50, 4.80) risk of acquiring depression. As WHO indicated depression occurs twice as frequently in women than in men (55). This result also agrees with a collaborative study carried out in 14 developed countries (56), and 21 developing countries (57) as well as independent studies in Ghana (58), Harar (Ethiopia) (29), and Ambo (Ethiopia) (59). Despite the complexity of sex differences in depression, recent evidence suggests that biological factors, particularly decreases in estrogen during menses, lactation, and menopause may contribute to the increased prevalence of depression in women. The majority of respondents in the current study found that the menopausal period verifies this reason. Estrogen decreases the release of GABA, increases the synthesis of dopamine and serotonin, and decreases their degradation and reuptake. Low estrogen level leads to a decline in well-being, sleep, mood, and cognitive functions (60). Identifying ligands that target the brain, such as estrogen receptor-β-selective ligands, may offer protection against depression (61). Similarly, because of X chromosomes have a higher density of genes related to the immune system, women mount stronger humoral and cellular immune responses than men for pathogen clearance. However, they become disadvantaged by higher expression of proinflammatory gene and cytokines and overall amplified systemic inflammation also can be a cause for depression (62).

In contrast, testosterone conversion in a male brain via aromatase, the presence of androgen receptors in hippocampal neurons, the non-recycling nature of testosterone in males, and the presence of sexually dimorphic brain nuclei in males provide unique protection against depression (63–66). Before we proceed with the conclusion, we want to underline that a reciprocal link between depression and its predictors may exist.

### Limitation of the study

We introduced the first baseline information in Ethiopia based on SII, a simple, cost-effective inflammatory marker. However, the design of the current study cannot establish causality. Additionally, the study did not investigate the effects or relationships of gut microbiota, particularly in the case of Irritable Bowel Syndrome (IBS), on inflammation and depression.

## Conclusions

This study revealed a high overall prevalence of depression among T2DM patients. Elevated SII, Khat chewing, and female sex were identified as risk factors for depression among T2DM patients. Therefore, healthcare providers should pay attention to the prevention, diagnosis, and treatment of depression among T2DM patients, especially among females. Additionally, a holistic approach that focuses on reducing systemic inflammation among patients with T2DM is recommended. Healthcare providers should also offer counseling and advice to T2DM patients to help them avoid risky behaviors such as Khat chewing. Lastly, we recommend future research to explore the relationship between Irritable Bowel Syndrome (IBS), inflammation, and depression in diabetes patients.

## Data Availability

The original contributions presented in the study are included in the article/supplementary material. Further inquiries can be directed to the corresponding author/s.
